# Isolation and Identification of β-Glucosidases-Producing Non-*Saccharomyces* Yeast Strains and Its Influence on the Aroma of Fermented Mango Juice

**DOI:** 10.3390/molecules28155890

**Published:** 2023-08-05

**Authors:** Yuemei Miao, Qiuping Zhong

**Affiliations:** 1 School of Food Science and Engineering, Hainan University, Haikou 570228, China; 20083200210023@hainanu.edu.cn; 2 Key Laboratory of Food Nutrition and Functional Food of Hainan Province, Haikou 570228, China

**Keywords:** mango juice, aroma, non-*Saccharomyces* yeast, β-glucosidases

## Abstract

The cultivation and enrichment of different soils in a vineyard yielded 95 yeast species. Among them, seven strains capable of producing β-glucosidases were identified using the aescin colorimetric method. One non-*Saccharomyces* yeast strain was isolated from a plate containing lysine and identified using internal transcription (ITS) as *Candida* cf. *sorbosivorans* (*C.* cf. *sorbosivorans*), which was named Candida cf. sorbosivorans X1. Additionally, the enzymatic characteristics of the β-glucosidases produced by this strain were investigated. The β-glucosidases generated by *C.* cf. *sorbosivorans* X1 displayed high enzymatic activity and enzyme-activity retention in a pH range of 3.0 to 5.4 and at temperatures of 30 °C to 35 °C. Using non-targeted metabolomics methods, we investigated the alterations in metabolites during the fermentation of mango juice. The strain *C.* cf. *sorbosivorans* X1 demonstrated activity against phenols and terpenes. In the fermented mango juice (X1FMJ), we identified 41 differential metabolites. These included 14 esters, 4 hydrocarbons, 3 aldehydes, 5 ketones, 4 terpenoids, 4 alcohols, 1 aromatic hydrocarbon, 2 amines, 1 acid, and 3 heterocyclic compounds. The metabolic pathways of these differential metabolites were analyzed, revealing four key pathways: tyrosine metabolism, phenylpropanoid biosynthesis, monoterpene biosynthesis, and α-linolenic acid metabolism, which promoted the formation of aroma compounds in the fermented mango juice.

## 1. Introduction

Mango (*Mangifera indica* L.), a fruit of significant importance in tropical and subtropical regions, belongs to the sumac family. It has delicate flesh, a unique taste, and abundant nutrients, making it highly favored by consumers. Fermented mango juice, with probiotic properties, has excellent market potential and development prospects. The aroma of mango is typically strong and pleasant, playing a crucial role in the sensory appeal of the juice and influencing consumers’ decisions to repurchase it [1].

To date, researchers have identified more than 600 volatile aroma compounds originating from more than 1000 distinct mango cultivars [2]. Quijano et al. investigated the volatile components of 9 Colombian mango varieties using simultaneous distillation extraction, GC, and GC-MS and identified 145 compounds, 8 of which were discovered for the first time in mango. The total concentration of volatile substances ranges between 17 and 75 mg/kg fresh weight. Terpenes are the main volatiles in all varieties, with 3-carene (Haden, Irwin, Manila, and Tommy Atkins), -pinene (Hilacha and Vallenato), phellandrene (Van Dyke), and terpinolene (Yulima) indicating the differences among the different mango varieties [3]. Munafo et al. isolated the aromatic active compounds from the mature fruits of five mango cultivars (Haden, White Alfonso, Praya Sowoy, Royal Special, and Malindi) using solvent extraction and solvent-assisted aroma evaporation, followed by GC-olfactory analysis. A total of 54 aromatic active compounds were compared and analyzed using aroma extract dilution, with flavor dilution (FD) factors ranging from 4 to 2048. Among these, 16 were reported in mangoes for the first time. The identification and FD factor results suggest that 4-hydroxy-2,5-dimethyl-3(2*H*)-furanone is a significant aromatic compound across all examined varieties. At least 27 aromatic active compounds with an FD factor of ≥128 are present in a single mango cultivar. The notable differences in FD factors of these odorants among the cultivars imply that they contribute to the distinct sensory characteristics of each cultivar [4]. The liberation and preservation of aroma compounds rely on their physicochemical characteristics and concentrations, while their detectability is affected by the interaction between the main juice constituents and the aroma compounds [5].

Prior to the liberation of aromatic substances, aroma compounds in their bound state need to undergo hydrolysis into free substances [6]. The use of non-Saccharomyces yeasts with high β-glucosidase production has been demonstrated for wine fermentation, which is crucial for the enhancement of aroma compounds, particularly terpenes and C13-pentadienes. The aroma of fermented wine is increased by the hydrolysis of glycoside-bound aroma compounds into free substances. Medium alkanol, enol, benzene derivatives, C13 isoprene, monoterpene, and sesquiterpene serve as flavor-active substances [7,8]. Terpenes, accounting for 16% to 90% of the identified mango aroma components, including α-pinene, β-pinene, β-laurine, limonene, brazilian, caryophyllene, α-phellandrene, γ-terpenes, isoterpenes, and β-caryophyllene, provide aroma precursors [3]. β-glucosidase plays a critical role in the release of volatile aromatic substances during the hydrolysis process of glycosidic-bound aromatic compounds and is a key rate-limiting enzyme in the synergistic effects with glycoside nucleic acid exonuclease [9]. The complexity of fermented product flavor is affected by the content and activity of the enzyme during fermentation. Most of the required β-glucosidases for hydrolysis come from raw materials and yeast, with the highest activity of β-glucosidases produced by non-*Saccharomyces* yeast. If the brewing yeast lacks β-glucosidase production or has insufficient enzyme activity, it can be supplemented to improve the complexity of the fermented wine aroma [10]. Spadaro et al. found that inoculation with the Metschnikowia pulcherrima strain activated β-d-glucosidase, leading to a significant increase in the content of *α*-terpineol, geraniol, and nerolidol. Numerous studies have highlighted the noteworthy influence of β-d-glucosidase activity produced by non-*Saccharomyces* yeast strains on the intricacy of wine aroma, particularly during the fermentation of fruit juices. Nevertheless, there are only a limited number of reports on the application of non-Saccharomyces yeast capable of producing glucosidase in mango juice fermentation [11].

Utilizing brewing yeast can enhance the aromatic components of the fermented mango juice, thereby improving its overall aroma. However, the carbon dioxide (CO_2_) produced by the brewing yeast during fermentation can significantly diminish the intrinsic mango aroma, resulting in a loss of the fruit’s unique scent in the final product [12]. Employing non-*Saccharomyces* yeast with a reduced fermentation capacity can help retain the distinctive aroma elements of the fruit, presenting a practical and effective approach to augment the scent of the fermented mango juice. Earlier research has explored the utilization of *Saccharomyces* yeast in the fermentation of mango juice and non-*Saccharomyces* yeast to enhance the aroma in wine and Baijiu. However, these studies concentrated mainly on the technological aspects, such as the combination of fermentation strains, conditions, inoculation ratios, and their effects on both volatile and non-volatile aroma constituents. Presently, there are no reports regarding the influence of the aroma compounds generated by non-*Saccharomyces* yeast that produce β-glucosidases on the mango juice aroma components during fermentation. Consequently, examining the aromatic components resulting from non-*Saccharomyces* yeast fermentation of mango juice and elucidating the mechanism of the alterations in the aroma constituents through the conversion of various aroma precursors by β-glucosidases during fermentation hold considerable practical importance for the production of aroma-coordinated probiotic mango juice products.

## 2. Results and Discussion

### 2.1. Screening of Non-Saccharomyces Yeasts

**Figure 1:**
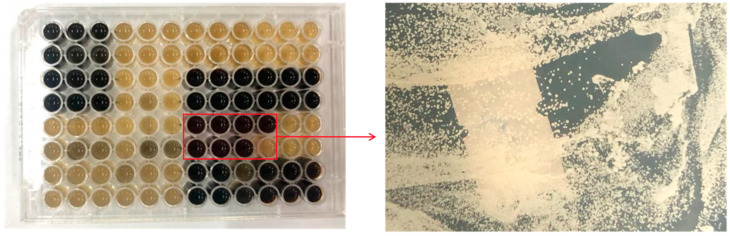
Chromogenic outcomes of primary screening in 96-well plates revealed that the formation of a black color with a phenolic iron complex indicates the production of β-glucosidase by the strain. Each 2 × 3 area on the 96-well plate represents a bacterial strain.

Ninety-five yeast strains were isolated from vineyard soil using the YPD medium. Each 2 × 3 area on the 96-well plate represented a bacterial strain (Figure 1). Seven strains producing β-glucosidase were chosen, and one non-*Saccharomyces* yeast strain (No.11, referred to as X1) was isolated from the lysine medium for subsequent molecular biology identification.

### 2.2. Molecular Biology Identification

**Figure 2:**
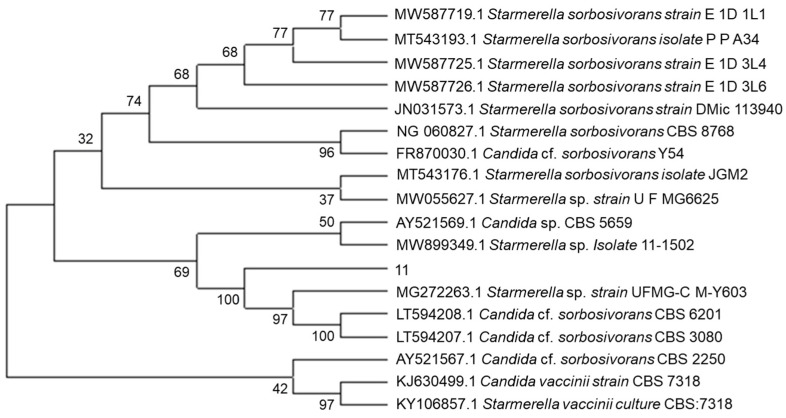
Phylogenetic tree of three yeast strains.

The phylogenetic tree was constructed based on ITS sequences (Figure 2). Sequence analysis revealed that X1 exhibited a 100% similarity to *Candida* cf. *sorbosivorans* CBS 6201 and CBS 3080, classifying it as *C.* cf. *sorbosivorans* X1. Wang et al. previously identified *Candida sorbosivorans* from fermented soy sauce and noted its positive impact on the aroma of the sauce [13]. Similarly, the selected strains in our study have been shown to enhance the aroma of fermented soy sauce. Given the resemblance in aroma production during glucose fermentation with yeast between fermented soy sauce and mango juice, we hypothesized that C. cf. sorbosivorans X1 could also enhance the aroma of mango juice fermentation. Consequently, this strain offers potential for investigating the aroma of fermented mango juice.

### 2.3. Enzymatic Properties of β-Glucosidase Produced by the Strain

The catalytic activity of enzymes can be affected by various factors, including environmental temperature, pH value, and substrate concentration [14]. Fruit juice fermentation environments exhibit distinctive features, such as low temperature and low pH. To ensure that the glycosidases function under the appropriate fermentation conditions and achieve optimal enzymatic activity, it is vital to investigate the adaptability of β-glucosidase produced by *C.* cf. *sorbosivorans* X1 in juice fermentation environments. Monitoring the glucose tolerance and ethanol tolerance of non-*Saccharomyces* yeast in the brewing environment is necessary. Belloh et al. observed the inhibitory effect of glucose on glycosidases. However, in this study, due to the low sugar content of the mango juice and the absence of ethanol production during the fermentation process, the glucose and ethanol tolerance of this strain was not analyzed [15]. When the *p*-nitrophenol content range was 0–0.6 mmol/L, the standard curve equation was y = 0.629x + 0.0679, with R^2^ = 0.9959, and a linear relationship between *p*-nitrophenol content and absorbance was observed.

**Figure 3:**
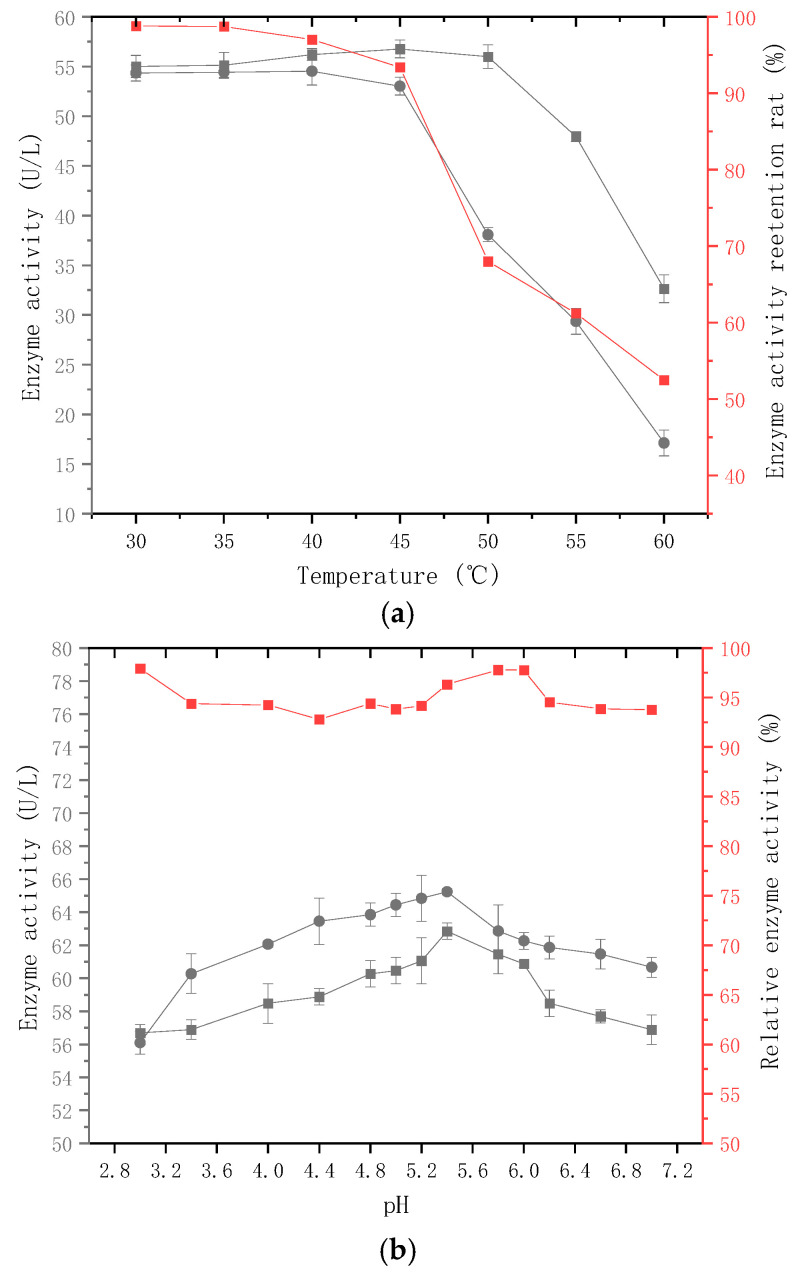
Effect of temperature and pH on enzyme activity. ● Enzyme activity at various reaction temperatures (**a**) and diverse pH values (**b**). ■ Enzyme activity stored in buffer at different temperatures (**a**) and pH levels (**b**) for 12 h. ■ Relative enzyme activity.

Temperature is an important factor influencing enzyme activity. Protein denaturation may occur at high temperatures, whereas low temperatures may affect the rate of enzyme catalysis. The *p*-NPG method was used to measure enzyme activity at different temperatures to investigate the effect of temperature on β-glucosidases activity. Figure 3a shows that when the temperature is between 30 °C and 50 °C, β-glucosidases enzyme activity is relatively high, peaking at 54.53 U/L at 40 °C. To investigate thermal stability, the highest enzyme activity retention rate was discovered at 30 °C to 35 °C after 40 min of storage in a water bath, ranging from 90% to 100%. The enzyme activity was significantly reduced after 40 min in the water bath at 40 °C to 60 °C.

**Figure 4:**
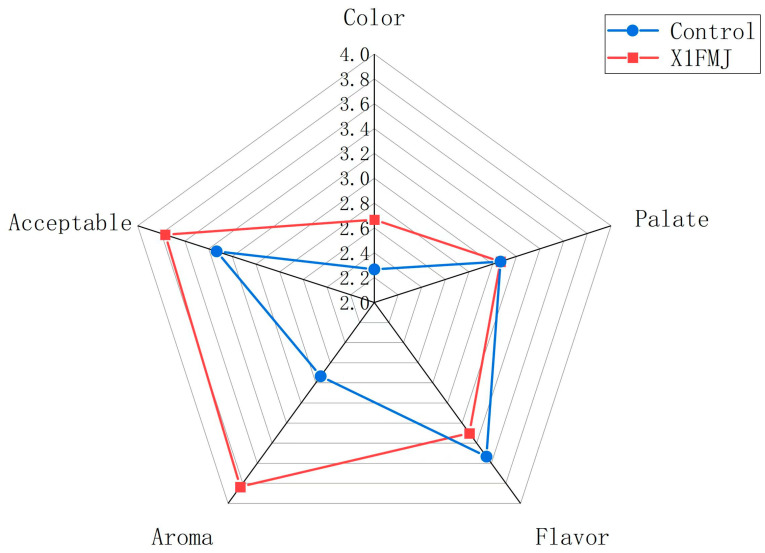
Sensory analysis of CONTROL and mango juice fermented by *C.* cf. *sorbosivorans* X1 (X1FMJ).

**Figure 5:**
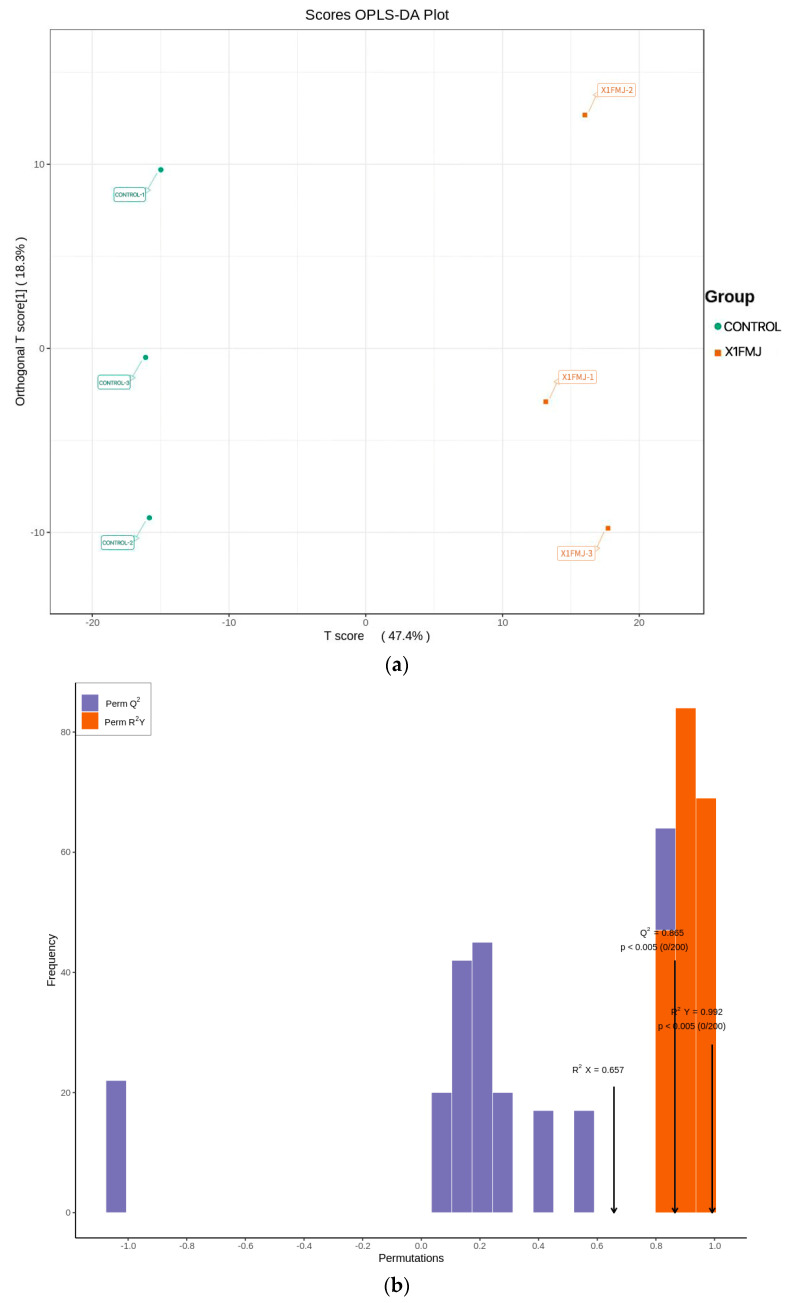
Results of OPLS−DA. (**a**) The x-axis represents the predicted principal component. The y−axis represents the orthogonal principal component, and the percentage indicates the component’s contribution to the dataset [1]. (**b**) OPLS−DA validation plot. The y-axis represents the frequency of the model’s classification effect in 200 random permutation combination experiments. The orange bars represent the random grouping model R^2^Y. The purple bars represent the random grouping model Q^2^, and the black arrows indicate the values of R^2^X, R^2^Y, and Q^2^ of the original model.

The pH value can impact the dissociation of particular functional groups in the protein’s spatial structure, resulting in changes in the protein conformation, subsequently affecting the enzyme activity. To investigate the effect of pH on β-glucosidases activity, the p-NPG method was utilized to determine the enzyme activity of β-glucosidases produced by *C.* cf. *sorbosivorans* X1 under varying pH conditions from 3.0 to 7.0, as depicted in Figure 3b. According to the results, the activity of glycosidase generated by *C.* cf. *sorbosivorans* X1 increased with pH values below 5.4 and decreased with pH values above 5.4. At pH 5.4, the enzyme activity reached its peak at 65.24 U/L, indicating that the optimal pH value for the reaction was 5.4. After maintaining the β-glucosidases solution produced by *C.* cf. *sorbosivorans* X1 in a 28 °C water bath at pH values ranging from 3.0 to 7.0 for 12 h, the enzyme activity was assessed using the *p*-NPG method, with the results displayed in Figure 3, Figure 4 and Figure 5. The stability of β-glucosidases was relatively high under pH values of 2.8–6.0, and the enzyme activity retention rate exceeded 90%, suggesting that β-glucosidases displayed robust enzymatic stability under acidic conditions. Consequently, the optimal pH value for enzyme catalysis lay between 2.8 and 6.0, and the optimal temperature ranged from 30 °C to 35 °C. The pH of mango juice typically hovers around 4.6. For this experiment, a fermentation temperature of 30 °C was selected, and the pH of the mango pulp was not manually adjusted, considering the heat sensitivity of the mango pulp, in order to maintain the quality of the fermented mango juice.

### 2.4. Sensory Analysis

Figure 4 presents the sensory evaluation of the control group (CONTROL) and X1FMJ. The sensory assessment of the mango juice encompassed five aspects: color, palate, flavor, aroma, and acceptability. In comparison with the control group, the taste of the fermentation group diminished, possibly due to a substantial reduction in soluble solids content during the fermentation process. Following fermentation, the overall acceptability, color, and aroma of the mango juice improved, but the flavor score decreased significantly. This decrease in flavor score could be attributed to the fermentation consuming the sugars and producing excessive acid, resulting in the juice becoming overly acidic. However, in terms of the sensory quality, the fermentation group still outperformed the control group overall.

### 2.5. Analysis of Aroma Composition by GC-MS

#### 2.5.1. Results of OPLS–DA

With the OPLS-DA model, samples from the control group and the fermentation group could be easily distinguished. As seen in Figure 5a, there was a significant difference in the metabolites between the control group and the fermentation group [16]. The OPLS-DA model was employed to detect whether the constructed model was “overfitting” through displacement tests. The essential parameters of the OPLS-DA model were anticipated to include R^2^X, R^2^Y, and Q^2^. R^2^X and R^2^Y indicate the model’s capacity to explain the X and Y matrices, respectively, while Q^2^ indicates the expected accuracy of the model. When these three indicators exceed 1, it indicates that the model’s security and reliability are high. If Q^2^ > 0.5, the model can be considered effective, and if Q^2^ > 0.9, it can be considered excellent. Figure 5b displays the OPLS-DA validation results for this experiment, with R^2^X = 0.657 and R^2^Y = 0.992, both close to 1, and Q^2^ = 0.865 > 0.5, suggesting that the model possesses high predictability.

#### 2.5.2. Analysis of Differential Metabolites

Table 1 displays the screening results of the differential volatile aromatic compounds in the fermentation and control groups. A total of 41 differential metabolites were identified, including 14 esters, 4 alcohols, 5 ketones, 3 heterocyclic compounds, 4 hydrocarbons, 4 terpenoids, 2 amines, 3 aldehydes, 1 aromatic hydrocarbon, and 1 acid. Table 2 presents the changes in the content of the differential metabolites. Scientific notation was utilized to express the content of each distinct metabolite.

**Figure 6:**
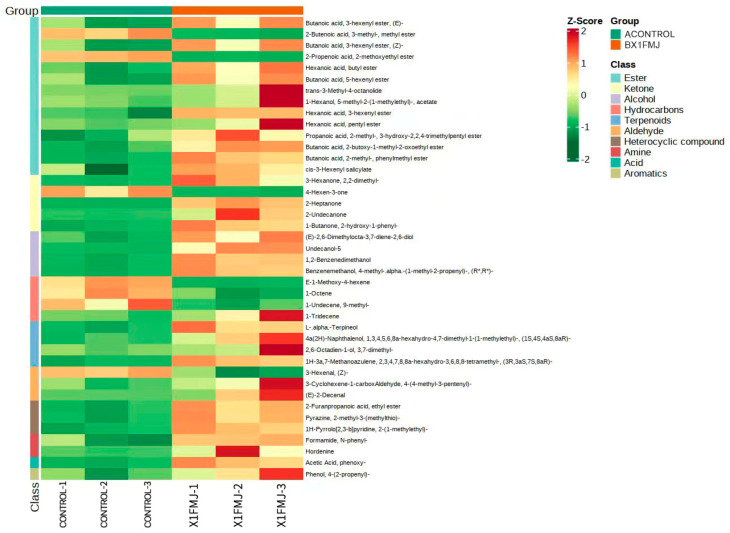
Heatmap analysis of CONTROL(before fermentation) and X1FMJ (after fermentation).

Figure 6 illustrates the heat map of the screened differential metabolites. Dark red signifies a high relative content of metabolites, while dark green indicates a low relative content. The larger the color difference, the more significant the difference in the metabolites. There were a total of seven downregulated differential metabolites, including two esters: 2-Butenoic acid-3-methyl-methyl ester and 2-Propenoic acid-2-methoxyethyl ester; three hydrocarbons: (*E*)-1-Methoxy-4-hexene, 1-Octene, and 1-Undecene, 9-methyl-; one ketone: 4-Hexen-3-one; and one aldehyde: 3-Hexenal, (*Z*)-. Out of these, methoxyethyl acrylate exhibited a subtle moldy odor, and its reduction contributed positively to the aroma of the fermented mango juice. A total of 34 differentially upregulated metabolites were identified, with 13 of them being the most significantly upregulated. These include four esters: Hexanoic acid, putyl ester, trans-3-Methyl-4-octanolide, 1-Hexanol, 5-methyl-2- (1-methylethyl)-, acetate, and Hexanoic acid, 3-hexenyl ester; one hydrocarbon: 1-Tridecadiene Hydrocarbons; one ketone: 2-Undecanone; two aldehydes: 3-Cyclohexene-1-carboxAldehyde, 4-(4-methyl-3-pentenyl)-,and (*E*)-2-Decenal; one amine: Hordenine; three terpenoids: L-.alpha.-Terpineol, 1,3,4,5,6,8a-hexahydro-4,7-dimethyl-1- (1-methylyl) -, 4a (2*H*)-Naphthalenol, (1*S*, 4*S*, 4a*S*, 8a*R*) -, 2,6-Octadien-1-ol, 3,7-dimethyl-; and one aromatic hydrocarbon: Phenol, 4-(2-propenyl)-. Stefani de Ovalle demonstrated that the strain of *I. terricola* BGL displayed significant specificity for norisoprene aglycone, such as 3-oxo-7,8-dihydrogen α-Violet alcohol and 3-oxo-α-Violet alcohol [17]. β-glucosidases exhibited the highest activity and specificity against various types of norisoprene, phenols, and terpenes [18]. Based on the differential metabolite screening results, it is speculated that strain X1, which produces β-glucosidases, exhibited activity towards hexene during the fermentation process. In terms of the ligand specificity, the X1 fermentation treatment showed considerable differences compared with CONTROL. The content of 3-Hexenal, (*Z*)- and 4-Hexen-3-one were significantly downregulated, while the upregulation of ketones without hexene, such as 2-Heptanone, 2-Undecanone, and 3-Hexanone, 2,2-dimethyl-, indicated that β-glucosidase facilitated the hydrolysis of hexene. In comparison with the control group, the fermentation group produced a higher quantity of esters with pleasant sweet, floral, and fruity flavors. These included Hexanoic acid, butyl ester (imparting pineapple and wine-like aroma), Hexanoic acid, pentyl ester (with apple and pineapple aroma), Butanoic acid, 2-butoxy-1-methyl-2-oxoethyl ester (providing a soft cream and toasted bread aroma), Hexanoic acid, 3-hexenyl ester (with a pear-like flavor), and *cis*-3-Hexenyl salicylate (releasing a rhododendron aroma). The group also produced more acids, such as Acetic Acid, phenoxy- (with a sweet–sour aroma and a honey-like taste); more ketones, such as 2-Undecanone (with a unique aroma similar to rutin and a peach-like aroma at low concentrations); more terpene compounds, such as l-alpha-Terpineol (clove aroma), 2,6-Octadien-1-ol, 3,7-dimethyl- (blooming rose flavor), 4a(2*H*)-Naphthalenol,1,3,4,5,6,8a-hexahydro-4,7-dimethyl-1-(1-methylethyl)-, (1*S*,4*S*,4a*S*,8a*R*)- (slightly sweet and medicinal cool aroma, resembling fresh and fragrant root medicinal cool aroma), and 1*H*-3a,7-Methanoazulene, 2,3,4,7,8,8a-hexahydro-3,6,8,8-tetramethyl-, (3*R*,3a*S*,7*S*,8a*R*)- (with a sweet and soft cypress wood characteristic aroma); more aldehydes, such as (*E*)-2-Decenal (with a strong rose and citrus aroma after dilution); and more aromatic hydrocarbons, such as Phenol, 4-(2-propenyl)- (slight pepper flavor). The content of Hordenine (with a pleasant and slightly irritating odor) was also significantly upregulated. These components can effectively enhance the aroma of fermented mango juice.

#### 2.5.3. Research on Differential Metabolite Metabolic Pathways 

Table 3 displays the metabolites, with the differences annotated in each pathway. Among all detected metabolites, 55 were annotated by KEGG, with 5 identified as differential metabolites. According to Table 2, three identified metabolites belonged to the alpha-Linolenic acid metabolism pathway, with 3-Hexenal, (*Z*)- being marked as a differential metabolite. Additionally, 18 identified metabolites were associated with the biosynthesis of secondary metabolites pathway, wherein l-alpha-Terpineol, 3-Hexenal, (*Z*)-, and Geraniol (2,6-Octadien-1-ol, 3,7-dimethyl-) were annotated as differential metabolites. Furthermore, six detected metabolites were linked to the Monoterpenoid biosynthesis pathway, featuring Geraniol and l-alpha-Terpineol as differential metabolites. Additionally, three identified metabolites were associated with Phenylpropanoid biosynthesis, with Phenol, 4-(2-propenyl)- marked as a differential metabolite. Lastly, three detected metabolites were annotated as Tyrosine metabolism, with maltine identified as a differential metabolite.

**Figure 7:**
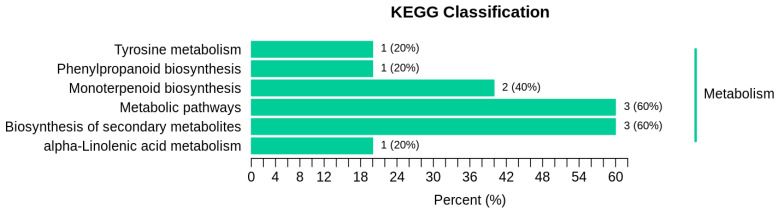
Differential metabolite KEGG classification diagram. The y-axis represents the name of the KEGG metabolic pathway, while the x-axis represents the number of differential metabolites annotated to the pathway and the ratio of differential metabolites annotated to the pathway to differential metabolites annotated to all pathways.

**Figure 8:**
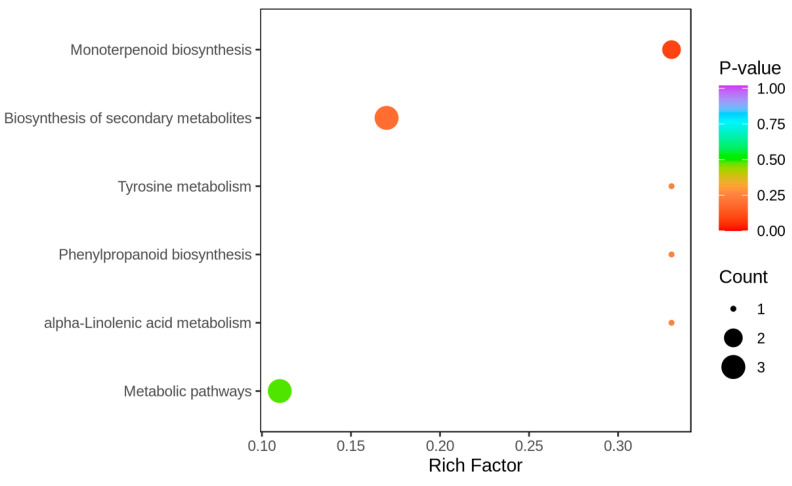
Differential metabolite KEGG enrichment map. The x-axis represents the Rich Factor corresponding to each channel. The y-axis displays the pathway names (arranged by *p*-value), with the dot color reflecting the magnitude of the *p*-value (redder indicates stronger enrichment). The size of the dots indicates the number of enriched differential metabolites.

As shown in Figure 7, three differential metabolites were annotated as the biosynthetic and metabolic pathways of the secondary metabolites, accounting for 60% of the total annotated metabolites. One differential metabolite was annotated to the α-linolenic acid metabolism pathway, accounting for 20% of the total annotated differential metabolites. Two differential metabolites were annotated in the biosynthesis pathway of monoterpenes, accounting for 40% of the total annotated differential metabolites. One differential metabolite of the phenylpropane biosynthesis pathway accounted for 20% of the total differential metabolites. One differential metabolite was annotated in tyrosine metabolism, accounting for 20% of the total annotated differential metabolites. Figure 8 illustrates that the enrichment of the differential metabolites had the most significant impact on monoterpenoid biosynthesis, and differential metabolites were most enriched in the biosynthesis of the secondary metabolites.

**Figure 9:**
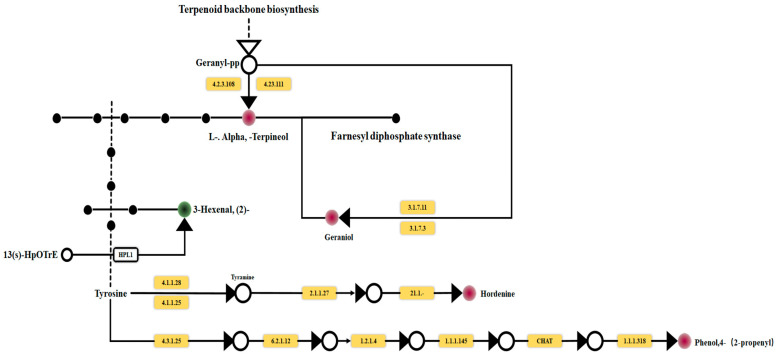
The metabolic pathway of differential metabolites in X1FMJ after fermentation. Green means the content of this metabolite downregulates and red means upregulates.

Figure 9, based on the integration of the differential metabolites’ positions annotated in each pathway within the corresponding metabolic pathway, illustrates the metabolic pathways of the differential metabolites under the influence of β-glucosidase. β-glucosidase hydrolyzed vanillin polypropylene in the mango juice, releasing the flavor precursor vanillin and promoting the synthesis of α-terpineol (with a purple clove aroma) and geraniol. San et al. discovered that β-d-glucosidase produced by the Metschnikowia pulcherima strain increased the concentration of Terpineol, Geraniol, and Nerolidol [19], which is in line with the findings of this study. Glycosidase enzymes can hydrolyze glycosidic-bound aroma compounds, releasing glucose and free aroma compounds. Both Terpineol and Nerol are derived from vanillin polypropylene in the linolenic acid metabolism pathway, representing a combination of vanillin glycoside and propylene. Given these findings, we can infer that the enzymatic hydrolysis of geranyl polypropylene led to the liberation of free vanillin, resulting in increased levels of terpineol and geraniol. Enzymes generated during microbial fermentation could catalyze the separation of the aromatic acids through hydrolysis. Tyrosine, an aromatic amino acid, was hydrolyzed by β-glucosidase during the fermentation process of mango juice, generating tyrosine residues. Subsequently, Hordenine was produced through Tyrosine metabolism, and Phenol, 4-(2-propenyl)- was formed via Phenylpropane biosynthesis.

## 3. Materials and Methods

### 3.1. Isolation and Prescreening of Non-Saccharomyces Yeasts

A soil sample (1 g), obtained from Renhe grape cultivation family farm in Jiangsu, China, was mixed with 9 mL of sterile water to create a suspension. Subsequently, 0.1 mL of bacterial solution was inoculated onto YPD agar medium (1% Yeast Extract, 2% Peptone, 2% Dextrose (glucose), 2% Agar) at 28 °C, and the culture was incubated for 72 h. Smooth milky-white circular strains were selected and inoculated in a YPD liquid culture medium, which was incubated at 28 °C and 180 r/min for 24 h. The subcultured samples were then uploaded onto YPD plates using the streak method and repeated three times to obtain single purified colonies.

The semi-quantitative colorimetric method was employed to conduct an initial screening of the strain’s β-glucosidase-producing ability. In this method, aescin was hydrolyzed to produce aescinate, which then reacted with Fe^3+^ and resulted in a black coloration. Following a three-day culture of the purified strain on a 96-well plate, the strain exhibiting a black color was identified as the one producing glycosidase. This strain was then inoculated onto a lysine medium, and purified strains that could grow on lysine plates were inoculated onto YPD agar slopes and stored at 4 °C for further research.

### 3.2. Molecular Biological Identification of Non-Saccharomyces Yeasts

To determine non-*Saccharomyces* yeast strains capable of producing glycosidases, their species identification was carried out based on the internal transcribed spacer (ITS) region [20]. The purified polymerase chain reaction (PCR) products of each strain were sequenced by the company (Sanshubio, Shanghai, China). The primer sequences used were 18sF AACTTAAAGGAATTGACGGAAG and 18sR TCCGCAGGTTCACCTACGGA. For this study, a BLAST search was conducted in the NCBI gene database, and a phylogenetic tree was constructed using the adjacency linking method in MEGAX [21].

### 3.3. Beta-Glucosidase Property Analysis

#### 3.3.1. Determination of Optimal Temperature and Thermal Stability of Enzymatic Reactions

Analysis of the enzymatic characteristics of β-glucosidase from chosen strains: Evaluation of β-glucosidase enzyme activity using the *p*-NPG method. Solutions of p-nitrophenol at varying concentrations (0.1, 0.3, and 0.5 mmol/L) were prepared, and 200 µL aliquots of each concentration were analyzed using a microplate reader. The absorbance was recorded at the maximum absorption wavelength of 415 nm to establish the standard curve for p-nitrophenol. A 5% inoculum of non-*Saccharomyces* yeast strains was introduced into the YPD medium and incubated for 24 h under shaking conditions at 28 °C and 180 r/min. Subsequently, 10% of the expanded non-*Saccharomyces* yeast solution was extracted and incorporated into the fermentation medium, which was incubated for 72 h under shaking conditions at 30 °C and 150 r/min. Upon completion of fermentation, the fermentation broth was subjected to centrifugation (8000 r/min) to acquire the crude enzyme solution. This crude enzyme solution was then combined with a citric acid–disodium hydrogen phosphate buffer at a pH of 5.5, to which 250 µL of 1 mmol/L 4-Nitrophenyl-alpha-d-galactopyranoside (*p*-NPG) was added. After 30 min, 1.0 mL of 1 mol/L Na_2_CO_3_ solution was introduced to terminate the reaction. The absorbance was measured at the maximum absorption wavelength for p-nitrophenol. Distilled water was employed as a blank control, and three replicates were performed for each sample. The enzyme activity unit (U) was defined as the amount of enzyme needed to catalyze the formation of 1 µmol of *p*-nitrophenol within 1 min at 40 °C and pH 4.5.

#### 3.3.2. Optimum Temperature and Thermal Stability of Enzyme Catalysis

The crude β-glucosidase enzyme solution was combined with a pH 6 buffer solution and exposed to varying temperatures (30 °C, 35 °C, 40 °C, 45 °C, 50 °C, 55 °C, and 60 °C) for a duration of 30 min. Enzyme activity was assessed using the *p*-NPG method to determine the optimal reaction temperature at which maximal activity was achieved. The crude enzyme solution was also subjected to a water bath for 40 min at the aforementioned temperatures. The enzyme’s thermal stability was precisely evaluated by comparing relative enzyme activities at different temperatures, with the activity of the unheated crude enzyme solution set as the 100% reference point.

#### 3.3.3. Optimum pH and pH Stability of Enzyme Catalysis

At the optimal reaction temperature, the crude β-glucosidase enzyme solution was introduced to a citric acid disodium hydrogen phosphate buffer solution with varying pH levels (3.0, 3.4, 4.0, 4.4, 4.8, 5.2, 6.0, 6.4, 6.8, and 7.2). Enzyme activity was assessed using the p-NPG method after a 30 min reaction, with the optimal pH being the one that showed the highest enzyme activity. The crude enzyme solution was mixed with buffer solutions of varying pH values and then incubated in a 28 °C water bath for 12 h. Subsequently, residual enzyme activity was measured, and relative enzyme activity was determined, using the activity without water bath preservation as the 100% reference to assess pH stability.

### 3.4. MJ Preparation and Fermentation

The mangoes used in this study were all mature Omang, sourced from a mango plantation in Haikou, Hainan, and free of significant mechanical damage. Mango juice was homogenized, portioned into 250 mL packages, and sterilized in an 88 °C water bath for 15 min. Next, 0.1% 108 CFU/mL of X1 was inoculated into the prepared mango juice and fermented at 30 °C for 72 h, referred to as the fermentation group. Mango juice without non-*Saccharomyces* yeast inoculation served as the control group. 

### 3.5. Sensory Assessment

Sensory evaluation was conducted following the method described by Zhang et al., with minor adjustments [18,22]. A total of 60 volunteers without color blindness, color weakness, or loss of taste or smell were recruited from Hainan University. These volunteers were regular or occasional consumers of mango juice. Fermented mango juice samples were collected after inoculation and compared with untreated mango juice for sensory evaluation, which was completed within 3 min. To prevent sensory fatigue, a 2 min rest was given during the evaluation, and drinking water was provided to cleanse the palate.

Sensory attributes, including color, palate, flavor, aroma, and overall acceptability of the mango juice, were rated on a scale. For color, a score of 2.0 represented a completely black color, 3.0 indicated a yellowish-brown color, and 4.0 denoted a bright yellow hue.

Regarding the palate, a score of 2.0 indicated a more astringent taste with higher liquid content, 3.0 represented a softer taste with increased liquid content, and 4.0 indicated a smoother taste with a better solid–liquid ratio.

For flavor, a score of 2.0 signified sourness at the entrance and a slightly bitter aftertaste, 3.0 indicated both sour and sweet tastes at the entrance with a subtle bitter aftertaste, and 4.0 represented a blend of sour and sweet tastes at the entrance with a pleasant, sweet aftertaste.

Regarding overall acceptability, a score of 2.0 indicated complete unacceptability and unwillingness to purchase, 3.0 represented acceptability without priority for purchasing, and 4.0 indicated strong liking and willingness to purchase.

Lastly, for aroma, a score of 2.0 indicated a faint and mixed aroma, 3.0 represented a pure but not very prominent aroma, and 4.0 denoted a clear and delightful aroma.

### 3.6. Volatile Aromatic Compound (VOC) Analysis

Five grams of FMJ were ground into a powder using liquid nitrogen and promptly transferred to a 20 mL headspace vial (Agilent, Palo Alto, CA, USA) containing a NaCl-saturated solution to prevent any enzyme reaction. The vials were sealed with crimp-top caps featuring TFE-silicone headspace septa (Agilent). For SPME analysis, each vial was placed at 60 °C for 5 min, and then a 120 µm DVB/CWR/PDMS fiber (Agilent) was exposed to the sample’s headspace for 15 min at 100 °C.

GC-MS conditions: Following sampling, the VOCs from the fiber coating were desorbed in the injection port of the GC apparatus (Model 8890, Agilent, CA, USA) at 250 °C for 5 min in splitless mode. The VOCs were identified and quantified using the Agilent Model 8890 GC and the 7000D mass spectrometer (Agilent, CA, USA), equipped with a 30 m × 0.25 mm × 0.25 µm DB-5MS (5% phenyl-polymethylsiloxane) capillary column. Helium served as the carrier gas, with a linear velocity of 1.2 mL/min. The injector and detector temperatures were maintained at 250 °C and 280 °C, respectively. The oven temperature was programmed from 40 °C (held for 3.5 min), increased by 10 °C/min to 100 °C, then by 7 °C/min to 180 °C, and finally by 25 °C/min to 280 °C, where it was held for 5 min. Mass spectra were recorded in electron impact (EI) ionization mode at 70 eV. The quadrupole mass detector, ion source, and transfer line temperatures were set at 150 °C, 230 °C, and 280 °C, respectively. The identification and quantification of analytes were carried out using the selected ion monitoring mode.

### 3.7. Differential Metabolite Screening

Variable importance in projection (VIP) values for metabolites were acquired from the model established using Orthogonal Partial Least Squares Discriminant Analysis (OPLS-DA). The raw data from OPLS-DA were log2-transformed and subsequently underwent centralized processing. In this process, X represented the sample quantitative information matrix, while Y denoted the sample grouping information matrix. The MetaboAnalystR package OPLSR.Anal function in R software (4.0.3) was employed for data analysis. Based on the OPLS-DA results, the VIP values from the obtained multivariate analysis OPLS-DA model enabled preliminary screening of metabolites with differences among distinct varieties or tissues. Concurrently, *p*-values or Fold Change values from the univariate analysis could be integrated to refine further the selection of differential metabolites. When biological replicates numbered less than 3, differential screening was conducted based on Fold Change values. If biological replicates were equal to or exceeded 3, a combination of VIP values and Fold Change from the OPLS-DA model was applied to screen differential metabolites. The screening criteria were as follows: 1. Metabolites with VIP ≥ 1 were selected. The VIP value signified the magnitude of the influence of inter-group differences for corresponding metabolites on the classification and discrimination of sample groups within the model. It is commonly accepted that metabolites with VIP ≥ 1 exhibit significant differences. 2. Metabolites with Fold Change ≥ 2 and Fold Change ≤ 0.5 were selected. If the disparity in metabolites between the control group and the experimental group exceeded 2 or was less than 0.5, the difference was deemed significant. 

### 3.8. KEGG Annotation and Enrichment Analysis

The identified metabolites were annotated using the KEGG compound database (http://www.kegg.jp/kegg/compound/ accessed on 7 July 2022). These annotated metabolites were subsequently mapped to the KEGG pathway database (http://www.kegg.jp/kegg/pathway.html accessed on 21 February 2023). The pathways containing significantly regulated metabolites were then subjected to metabolite set enrichment analysis, and their significance was assessed using the p-values obtained from the hypergeometric test.

### 3.9. Data Processing

The data processing techniques employed in all metabolomic analysis procedures comprised primarily UV (unit variance scaling) and Ctr (zero-centered). Origin 8.0 software (OriginLab, Northampton, MA, USA) was used for drawing.

(1) UV (unit variance scaling), also known as Z-score standardization or auto-scaling, is a method for standardizing data based on the mean and standard deviation of the original data. The processed data adhere to a standard normal distribution, meaning that the mean is 0 and the standard deviation is 1. The calculation method involves dividing by the variable’s standard deviation after data centralization, using the following formula: x’ = (x – µ)/σ, where µ represents the mean and σ signifies the standard deviation.
(2) Zero-centered calculation method. The means of variables are subtracted from the original data, using the following formula: x’ = x − µ, where µ is the mean.

## 4. Conclusions

Non-*Saccharomyces* yeast strains that produce aroma were screened from the soil of orchards where grapes were cultivated year-round. Preliminary screening was conducted using the WLN plate growth and aescin colorimetric methods to select the yeast strains capable of producing β-glucosidase. Non-*Saccharomyces* yeast was chosen using the lysine plate growth method for molecular biology identification. The optimal conditions for mango juice fermentation were determined by measuring the enzyme activity of the screened strains capable of producing the enzyme. Under these optimal conditions, mango juice fermentation was carried out, and aroma components and metabolic pathways were analyzed. The primary conclusions are as follows:

Ninety-five yeast species were obtained by enriching and cultivating the orchard soil. Using the aescin colorimetric method, seven yeast strains that produce β-glucosidases were isolated, while a lysine-containing plate was used to isolate one non-*Saccharomyces* yeast strain. A strain (X1) exhibiting glycosidase activity was identified as *Candida* cf. *sorbosivorans* X1 through internal transcribed spacer (ITS) sequencing. A strain (X1) with glycosidase activity was screened and identified by internal transcription (ITS) as *C.* cf. *sorbosivorans*, named *Candida* cf. *sorbosivorans* X1. The β-glucosidase produced by the strain exhibited the highest enzyme activity at a pH of 5.4, with 65.24 U/L. At a temperature of 40 °C, the highest enzyme activity was 54.53 U/L, and the optimal fermentation condition was 30 °C, without changing the pH of the original slurry. 

After analyzing the aroma components of the mango juice before and after fermentation, it was discovered that *C.* cf. *sorbosivorans* X1 had high activity on the precursor geranyl polypropylene of α-terpineol bound with a glycosidic bond. This process was able to hydrolyze the geranyl polypropylene present in the mango juice, resulting in the release of the flavor precursor geranyl and facilitating the synthesis of α-terpineol (providing a purple clove flavor) and geraniol. Additionally, *C.* cf. *sorbosivorans* X1 was capable of hydrolyzing tyrosine to produce tyrosine residues, which then underwent further transformation through tyrosine metabolism to obtain maltine. During this metabolic pathway, Phenol, 4-(2-propenyl)- was produced via phenylpropane biosynthesis. Simultaneously, more esters, acids, and aromatic hydrocarbons were generated during the fermentation process, effectively improving the aroma and quality of fermented mango juice.

## Figures and Tables

**Table 1:** Differential metabolite screening results.

Index	Compounds	Class I
XMW1069	Butanoic acid, 3-hexenyl ester, (*E*)-	Ester
XMW0881	2-Butenoic acid, 3-methyl-, methyl ester	Ester
KMW0366	Butanoic acid, 3-hexenyl ester, (*Z*)-	Ester
XMW0180	2-Propenoic acid, 2-methoxyethyl ester	Ester
KMW0381	Hexanoic acid, butyl ester	Ester
XMW0304	Butanoic acid, 5-hexenyl ester	Ester
KMW0490	trans-3-Methyl-4-octanolide	Ester
D457	1-Hexanol, 5-methyl-2-(1-methylethyl)-, acetate	Ester
XMW1097	Hexanoic acid, 3-hexenyl ester	Ester
XMW1244	Hexanoic acid, pentyl ester	Ester
XMW0617	Propanoic acid, 2-methyl-, 3-hydroxy-2,2,4-trimethylpentyl ester	Ester
XMW0962	Butanoic acid, 2-methyl-, phenylmethyl ester	Ester
w44	Butanoic acid, 2-butoxy-1-methyl-2-oxoethyl ester	Ester
XMW0479	*cis*-3-Hexenyl salicylate	Ester
D422	Undecanol-5	Alcohol
XMW0443	(*E*)-2,6-Dimethylocta-3,7-diene-2,6-diol	Alcohol
XMW0107	1,2-Benzenedimethanol	Alcohol
XMW0728	Benzenemethanol, 4-methyl-. alpha.-(1-methyl-2-propenyl)-, (R*, R*)-	Alcohol
XMW0648	3-Hexanone, 2,2-dimethyl-	Ketone
XMW0201	4-Hexen-3-one	Ketone
KMW0113	2-Heptanone	Ketone
KMW0474	2-Undecanone	Ketone
XMW0856	1-Butanone, 2-hydroxy-1-phenyl-	Ketone
D81	2-Furanpropanoic acid, ethyl ester	Heterocyclic compound
KMW0068	3-Hexenal, (*Z*)-	Heterocyclic compound
D323	Pyrazine, 2-methyl-3-(methylthio)-	Heterocyclic compound
XMW0701	1*H*-Pyrrolo [2,3-b] pyridine, 2-(1-methylethyl)-	Heterocyclic compound
XMW0279	*(E)*-1-Methoxy-4-hexene	Hydrocarbons
XMW0775	1-Undecene, 9-methyl-	Hydrocarbons
XMW1114	1-Octene	Hydrocarbons
D266	1-Tridecene	Hydrocarbons
NMW0071	l-alpha-Terpineol	Terpenoids
XMW0854	4a(2*H*)-Naphthalenol,1,3,4,5,6,8a-hexahydro-4,7-dimethyl-1-(1-methylethyl)-, (1*S*,4*S*,4a*S*,8a*R*)-	Terpenoids
NMW0104	2,6-Octadien-1-ol, 3,7-dimethyl-	Terpenoids
XMW1412	1*H*-3a,7-Methanoazulene, 2,3,4,7,8,8a-hexahydro-3,6,8,8-tetramethyl-, (3*R*,3a*S*,7*S*,8a*R*)-	Terpenoids
XMW0711	Formamide, *N*-phenyl-	Amine
NMW0218	Hordenine	Amine
GMW0087	(*E*)-2-Decenal	Aldehyde
D425	3-Cyclohexene-1-carboxAldehyde,4-(4-methyl-3-pentenyl)-	Aldehyde
KMW0433	Phenol, 4-(2-propenyl)-	Aromatics
NMW0171	Acetic Acid, phenoxy-	Acid

**Table 2:** Differential metabolites of CONTROL and X1FMJ.

Index	Content (µg/L × 10^3^)		VIP	*p*-Value	Type	Fold Change	Cpd ID	KEGG Map
	Control	X1FMJ						
XMW1069	5.98 × 10^5^	1.72 × 10^6^	1.28	1.49 × 10^−2^	up	2.87	-	-
XMW0881	8.52 × 10^5^	1.45 × 10^5^	1.45	2.17 × 10^−3^	down	1.71 × 10^−1^	-	-
KMW0366	6.24 × 10^5^	1.79 × 10^6^	1.28	1.54 × 10^−2^	up	2.87	-	-
XMW0180	3.22 × 10^5^	3.53 × 10^4^	1.44	4.40 × 10^−4^	down	1.10 × 10^−1^	-	-
KMW0381	2.62 × 10^4^	5.64 × 10^4^	1.36	1.59 × 10^−2^	up	2.15	-	-
XMW0304	3.26 × 10^5^	9.58 × 10^5^	1.28	1.53 × 10^−2^	up	2.93	-	-
KMW0490	1.56 × 10^4^	4.76 × 10^4^	1.12	2.83 × 10^−1^	up	3.06	-	-
D457	1.59 × 10^4^	4.87 × 10^4^	1.06	3.03 × 10^−1^	up	3.07	D88387	-
XMW1097	1.97 × 10^4^	4.21 × 10^4^	1.37	1.13 × 10^−2^	up	2.14	-	-
XMW1244	1.45 × 10^4^	4.28 × 10^4^	1.14	2.56 × 10^−1^	up	2.96	-	-
XMW0617	6.74 × 10^4^	1.45 × 10^5^	1.24	2.83 × 10^−2^	up	2.14	-	-
XMW0962	5.90 × 10^4^	2.18 × 10^5^	1.42	3.14 × 10^−3^	up	3.69	-	-
w44	2.50 × 10^4^	6.71 × 10^4^	1.41	6.91 × 10^−3^	up	2.68	-	-
XMW0479	2.13 × 10^4^	5.43 × 10^4^	1.11	3.60 × 10^−2^	up	2.55	-	-
XMW0648	3.36 × 10^4^	1.89 × 10^5^	1.38	4.19 × 10^−2^	up	5.64	-	-
XMW0201	5.12 × 10^5^	4.67 × 10^4^	1.44	1.18 × 10^−2^	down	9.11 × 10^−2^	-	-
KMW0113	9.00	4.86 × 10^5^	1.45	2.89 × 10^−3^	up	5.40 × 10^4^	C08380	-
KMW0474	2.97 × 10^3^	2.12 × 10^5^	1.41	9.32 × 10^−2^	up	7.15 × 10	C01875	-
XMW0856	9.26 × 10^4^	3.85 × 10^5^	1.41	5.58 × 10^−3^	up	4.16	-	-
D81	4.76 × 10^4^	9.77 × 10^4^	1.41	1.30 × 10^−3^	up	2.05	-	-
KMW0068	4.27 × 10^4^	1.75 × 10^4^	1.36	1.52 × 10^−2^	down	4.09 × 10^−1^	C16310	ko00592, ko01110
D323	2.23 × 10^4^	4.60 × 10^4^	1.41	1.74 × 10^−3^	up	2.06	-	-
XMW0701	1.76 × 10^4^	4.26 × 10^4^	1.41	4.59 × 10^−4^	up	2.42	-	-
XMW0279	7.06 × 10^4^	8.42 × 10^3^	1.44	7.09 × 10^−3^	down	1.19 × 10^−1^	-	-
XMW0775	2.35 × 10^4^	6.56 × 10^3^	1.33	3.53 × 10^−2^	down	2.79 × 10^−1^	-	-
XMW1114	8.26 × 10^4^	3.79 × 10^4^	1.39	3.27 × 10^−3^	down	4.59 × 10^−1^	D91846	-
D266	6.57 × 10^3^	2.82 × 10^4^	1.25	1.83 × 10^−1^	up	4.29	D92374	-
NMW0071	1.39 × 10^5^	3.29 × 10^5^	1.40	8.80 × 10^−3^	up	2.37	C11393	ko00902, ko01100, ko01110
XMW0854	7.73 × 10^3^	1.82 × 10^4^	1.36	8.41 × 10^−2^	up	2.35	-	-
NMW0104	2.73 × 10^4^	7.42 × 10^4^	1.07	2.96 × 10^−1^	up	2.71	C01500	ko00902, ko01100, ko01110
XMW0711	1.87 × 10^4^	4.66 × 10^4^	1.30	3.10 × 10^−2^	up	2.49	D70279	-
NMW0218	1.09 × 10^5^	2.30 × 10^5^	1.27	1.37 × 10^−1^	up	2.12	C06199	ko00350, ko01100
GMW0087	1.50 × 10^4^	1.28 × 10^5^	1.28	1.75 × 10^−1^	up	8.53	-	-
D425	1.65 × 10^4^	3.57 × 10^4^	1.20	1.78 × 10^−1^	up	2.17	-	-
KMW0433	3.29 × 10^4^	7.75 × 10^4^	1.25	7.30 × 10^−2^	up	2.36	C16930	ko00940
NMW0171	3.33 × 10^4^	1.22 × 10^5^	1.42	2.91 × 10^−3^	up	3.67	C02181	-
XMW0775	2.35 × 10^4^	6.56 × 10^3^	1.33	3.53 × 10^−2^	down	2.79 × 10^4^	-	-
XMW0443	5.44 × 10^4^	1.19 × 10^5^	1.38	1.88 × 10^−2^	up	2.19	-	-
XMW0107	4.93 × 10^4^	2.26 × 10^5^	1.43	2.13 × 10^−3^	up	4.58	-	-
XMW0728	2.53 × 10^5^	1.21 × 10^6^	1.42	2.92 × 10^−3^	up	4.80	-	-
D422	1.14 × 10^4^	9.71 × 10^4^	1.45	2.44 × 10^−2^	up	8.52	-	-
XMW1412	6.72 × 10^4^	1.72 × 10^5^	1.42	8.88 × 10^−4^	up	2.56	-	-

VIP: Variable importance projection, *p*-value: Significance test *p*-value, Fold Change: Difference multiple.

**Table 3:** Statistical table of different metabolites for each pathway in KEGG.

KEGG_Pathway	ko_ID	Sig_Compound	Compound	Sig_Compound_All	Compound_All
alpha-Linolenic acid metabolism	ko00592	1	3	5	55
Biosynthesis of secondary metabolites	ko01110	3	18	5	55
Monoterpenoid biosynthesis	ko00902	2	6	5	55
Metabolic pathways	ko01100	3	27	5	55
Phenylpropanoid biosynthesis	ko00940	1	3	5	55
Tyrosine metabolism	ko00350	1	3	5	55

KEGG_pathway: name of the channel, ko_ID: ko number of the pathway in the KEGG database, Sig_compound: number of differential significant metabolites annotated by KEGG in this pathway, Compound: number of metabolites belonging to this pathway among the detected metabolites, Sig_compound_all: KEGG annotated to the difference in the number of significant metabolites, Compound_all: number of metabolites annotated by KEGG in all detected metabolites.

## Data Availability

The data presented in this study are available on request from the corresponding authors.
